# Ethnobotany, phytochemistry, pharmacology and toxicity of *Hagenia abyssinica* (Bruce) J.F.Gmel.: a review

**DOI:** 10.3389/fphar.2025.1593724

**Published:** 2025-06-26

**Authors:** Guohui Shi, Linlin Ni, Yang Zhang, Zhizi Qu, Xiaoni Kong, Honglei Zhou, Yan Xu

**Affiliations:** ^1^ Medical School, Shandong Xiehe University, Jinan, China; ^2^ College of Pharmacy, Shandong University of Traditional Chinese Medicine, Jinan, China; ^3^ The Second Affiliated Hospital of Shandong University of Traditional Chinese Medicine, Jinan, China; ^4^ College of Pharmacy, Shandong College of Traditional Chinese Medicine, Yantai, Shandong, China

**Keywords:** *Hagenia abyssinica* (Bruce) J.F.Gmel., traditional uses, phytochemistry, pharmacology, toxicity

## Abstract

*Hagenia abyssinica*: (Bruce) J.F.Gmel. (*H. abyssinica*), commonly known as “kosso,” is a prevalent phytomedicine addressing aliments across Ethiopia. Traditionally, it is used to treat fever, abdominal pain, respiratory illnesses, intestinal parasitic infections, wound healing, and cancer. Current research has revealed its anthelmintic, antimicrobial, antihyperglycemic, antidyslipidemic, antidiarrheal, and antioxidant activities. These pharmacological properties might be attributed to the presence of secondary metabolites, such as terpenoids, flavonoids, and phenols. Its crude extract is safe *in vivo* at doses less than 2,000 mg/kg, but larger doses can cause damage to the optic nerve. Although significant research findings are available, challenges remain. This paper provides a comprehensive review of research findings, identifying gaps in mechanistic studies and standardized toxicology assessments. Future priorities include applying ConPhyMP guidelines for extract characterization and integrating traditional knowledge with biodiversity conservation.

## Graphical Abstract

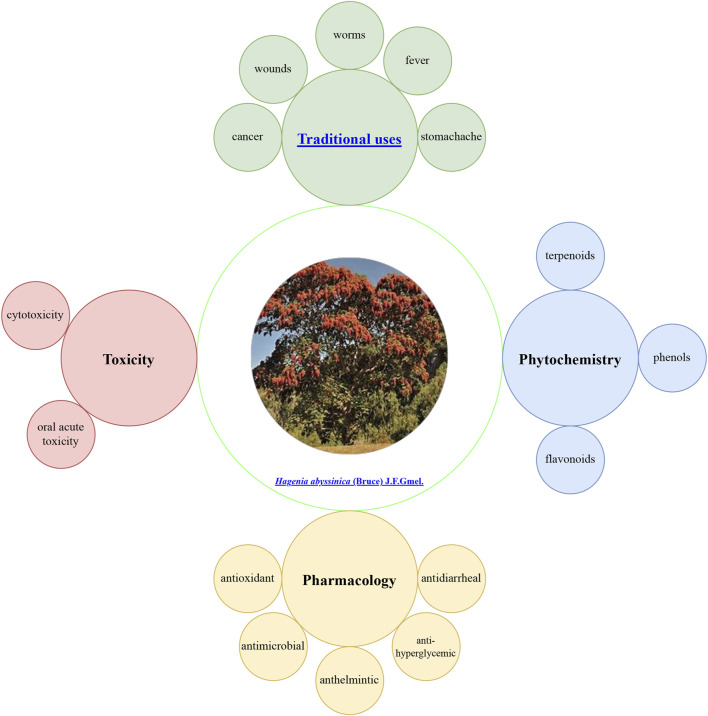

## 1 Introduction

Traditional medicines are vital to global health, particularly in regions lacking modern medical resources (Abdullahi, 2011; Emeje et al., 2023). Traditional uses are typically rooted in knowledge and cultural traditions that are transmitted orally across generations, closely tied to the local environment, culture, and belief systems (Staub et al., 2015). A significant portion of the population in rural and low-income urban areas depends on traditional medicine for primary healthcare, with phytotherapy being a predominant treatment method (Benoit-Vical, 2005; Coals et al., 2024). Africa, with its abundant natural medicines and rich biodiversity, holds significant potential for developing treatments for endemic diseases (Boukandou et al., 2015; Gruca et al., 2015). Notably, over 80 percent of the African population relies on traditional medicines, which are naturally sourced and often gathered locally (Dehyab et al., 2020; Frimpong et al., 2021; Moroole et al., 2019). Ethiopia, recognized as one of Africa’s six plant-rich nations, boasts 60% of its flora with medicinal properties (Ayalew et al., 2022; Gichira et al., 2017).

*H. abyssinica*, an important medicinal plant native to Africa, enjoys widespread acceptance and popularity in traditional medicine (Ayele et al., 2017; Bekele and Reddy, 2015). Traditionally, it is utilized for treating fever, stomachache, cold, intestinal worms, wound healing, cancer, and various other ailments (Tolossa, 2021; Yirga et al., 2022). Economically, it supports local communities through its applications in timber, furniture, house construction, and firewood, while its dried flowers command high market prices (Assefa et al., 2010). In the regions of Bale and Kofele, it is referred to as “hangefa muka,” denoting its status as one of the oldest and most esteemed trees (Assefa et al., 2010). However, increased market demand has led to overharvesting and grazing, resulting in a significant population decline. Consequently, *H. abyssinica* is now classified as an endangered species (Gichira et al., 2016).

Modern medicine increasingly recognizes the significance of *H. abyssinica*, offering valuable insights into its development. The chemical constituents and pharmacological properties of *H. abyssinica* have been prominent research topics. Phytochemical investigations have identified key metabolites, such as terpenoids, flavonoids, and phenols (Agidew, 2022; Wolde et al., 2016), which might be responsible for pharmacological effects, including anthelmintic, antimicrobial, antihyperglycemic, antidyslipidemic, antidiarrheal, and antioxidant activities(Alemu et al., 2018; Kifle and Belayneh, 2020; Murthy et al., 2020). Therefore, previous research provides a theoretical foundation for enhanced understanding and application of *H. abyssinica*.

The medicinal significance of *H. abyssinica* is notable, yet a comprehensive review is currently lacking. Given that, a systematic search was performed following PRISMA guidelines across Google Scholar, Web of Science, Sci-finder, PubMed, Elsevier, Wiley, China National Knowledge Infrastructure, Open Access Library, and SpringerLink using keywords: “*H. abyssinica*,” “kosso,” “ethnobotany,” “phytochemistry,” “pharmacology,” “toxicity”. Inclusion criteria: peer-reviewed preclinical studies; ethnobotanical surveys with quantitative data; toxicity assessments with LD_50_ values. Exclusion criteria: Non-english articles; case reports without controls. Of 378 identified articles, 68 met inclusion criteria after screening titles/abstracts. This systematic review addresses the following PICOS-defined research question: Population: preclinical studies (*in vitro*/*in vivo* models) and ethnobotanical reports; Intervention: phytochemical constituents and pharmacological activities of *H. abyssinica* extracts; Comparison: different extraction methods (crude vs. nanoparticle formulations), solvent polarities, and dosage variations; Outcomes: anthelmintic efficacy (parasite mortality rate), antimicrobial activity (inhibition zone), metabolic effects (blood glucose reduction), and safety profiles (LD_50_). Study design: preclinical experimental studies and ethnopharmacological surveys published between as of 2025.

This review consolidates research findings on *H. abyssinica*, while also exploring prospective research avenues. It is anticipated that this review will engage scholars and encourage further detailed investigations into *H. abyssinica*.

## 2 Ethnobotany

### 2.1 Botanical characteristics

**FIGURE 1:**
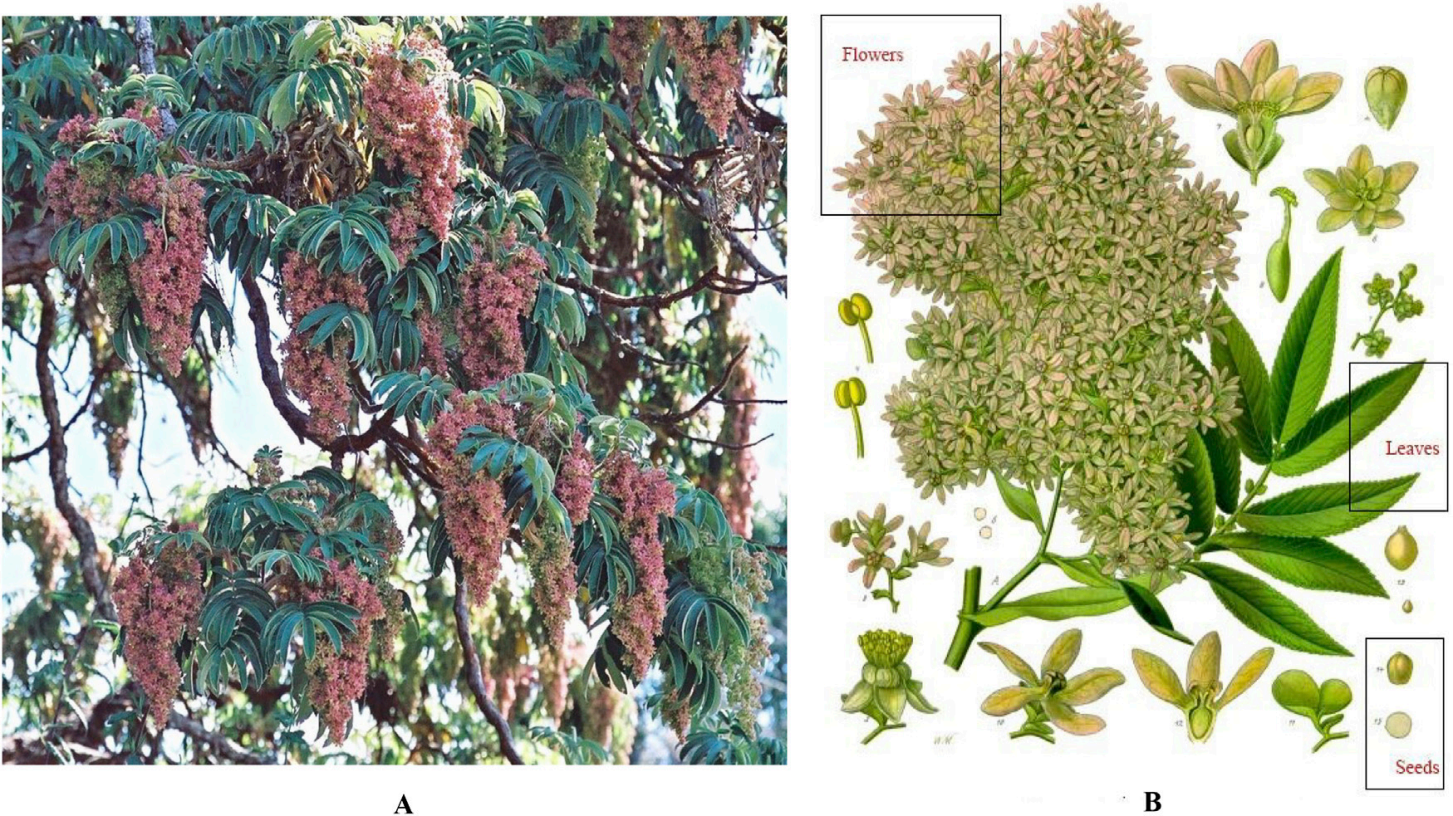
Botanical features of *H. abyssinica*. **(A)**: Full view; **(B)**: Plant parts (flowers, leaves and seeds). Images from the Useful Tropical Plants Database under creative commons license).

*H. abyssinica* is a tree of the Rosaceae family distributed in the East African highlands, with unique advantages in terms of species diversity and ecological adaptability. It is a tree up to more than 20 m high with a short trunk and thick, thin bark (https://tropical.theferns.info). The compound leaves have 7–13 leaflets, each of which has a finely serrated edge, and the leaves are green with a silvery-white tomentum covering the back of the blade (www.worldfloraonline.org). Its inflorescences can be 30–60 cm long, and the flowers come in a variety of colors such as white, orange-yellow, and pink (www.worldfloraonline.org). The flowers are dioecious, with the female flowers being widely utilized for their anthelmintic medicinal value (Figure 1).

*H. abyssinica* is mainly found in Ethiopia, Kenya, Uganda and other sub-alpine areas (2,000–3,000 m). It prefers moist and slightly cool, and grows mixed with *Podocarpus* and *Juniperus* procera, or forms dominant pure forests in arid areas (https://www.wikipedia.org). At low altitudes, it coexists with *Lobelia bambuseti*, constituting a special vegetation pattern (Bekele and Reddy, 2015). *H. abyssinica* has a strong ability to survive, and can reflect high-altitude ultraviolet light through the peeling of the bark against pests and diseases, as well as the downy hairs on the underside of the leaves (Assefa et al., 2010). *H. abyssinica* is mainly propagated by wind and birds, with lepidopterous insects playing an important role.

### 2.2 Traditional uses

*H. abyssinica*, an endemic tree species of the high-altitude regions of East Africa, occupies a central place in the traditional medicine system of Ethiopia and neighboring countries (Woldeamanuel et al., 2022). It is recognized by various names across regions, referring as “heto” in Kofele and Bale, and “kosso” in Debark (Assefa et al., 2010). Its medicinal history dates back hundreds of years and it is still the natural medicine of choice in many rural communities to fight parasitic infections and common diseases. Its bark, flower, root, leaf, and wood are utilized for medicinal purposes to address a range of ailments, including fever, stomachache, cold, intestinal worms, wound healing, cancer, cough, and diseases in livestock (Belachew et al., 2020; Fan et al., 2020; Karumi et al., 2013).

To enhance the healing properties of the plant, the local community has developed various applications (Assefa et al., 2010; Belete and Dagme, 2022; Yirga et al., 2022). In the Debark region of northern Ethiopia, traditional healers use female flowers to expel tapeworms from humans. This is done by crushing dried female flowers into a powder and adding honey, bananas, or a local fermented drink to make an oral suspension or paste. The preparation not only masks its bitter taste but is fermented to improve its efficacy, and patients usually drink it in the morning on an empty stomach and see the worms fall off in 4–6 h. In the southeastern Kofele and Bale regions, the female flowers are also used in combination with garlic and Artemisia leaves to fight malaria. If only the bark is used, it can help to reduce fever and alleviate coughs. It is worth noting that the inhabitants of Bale have invented a special fermentation process which mixes powdered female flowers with pumpkin seeds and cabbage leaves and allows them to rest for a few days, which reduces digestive side effects. The red juice of *H. abyssinica* is eaten raw to reduce abdominal pain, while the fresh leaves applied externally accelerate the healing of broken bones in domestic animals and are a popular treatment in northern pastoralist areas.

For pregnant women, a low dose of female pollen mixed with barley porridge paste is taken in many regions during the middle and late stages of pregnancy. This use is said to relieve maternal suffering and promote the health of both mother and fetus. To prevent miscarriages, doctors strictly control the dosage (0.5 g/kg for adults, halved for pregnant women). In the Abaya region of Oromia, locals commonly use H. abyssinica dried fruits with honey and Tella wine as a traditional treatment for roundworms. Among them, the decoction of H. abyssinica seeds is considered to be a commonly used broad-spectrum anthelmintic and is often combined with milk and water to improve palatability.

*H. abyssinica* is advised for consumption in the morning on an empty stomach to maximize its effectiveness (Bekele and Reddy, 2015). However, it frequently induces nausea, stomach discomfort, and persistent diarrhea, which are often regarded as normal by locals due to the absence of methods to mitigate these adverse effects. Consequently, there has been a decline in its consumption. It is worth mentioning that a variety of innovative modifications are implemented in the preparation techniques (Bekele and Reddy, 2015). For instance, the pollen is carefully cooked to create a unique and flavorful tea. This process not only enhances the taste but also preserved the beneficial properties of the pollen, making it a delightful and healthy beverage. It is recommended that patients avoid consuming beer, as it reduces the efficacy of the medication (Assefa et al., 2010). To minimize toxic side effects and improve patient adherence, there is a critical need to enhance the traditional application of *H. abyssinica*. The traditional uses of *H. abyssinica* were summarized in Table 1.

**TABLE 1:** Traditional uses of *H. abyssinica*.

Region	Plant part	Primary use	Compatibility materials	Dosage form	References
Debark (Northern Ethiopia)	Female flowers	Expel tapeworms (*Taenia saginata*)	Honey, banana, Tella (local beer)	Oral suspension/paste	Assefa et al. (2010)
Kofele (Southeastern Ethiopia)	Female flowers	Treat intestinal parasites, malaria adjuvant	Garlic (*Allium sativum*), Artemisia afra leaves	Decoction	
Bale (Southeastern Ethiopia)	Bark	Reduce fever, relieve cough	None	Decoction	
Bale (Southeastern Ethiopia)	Female flowers	Expel tapeworms	Pumpkin seeds (*Cucurbita pepo*), cabbage leaves	Fermented liquid	
Bale (Southeastern Ethiopia)	Sapwood liquid	Relieve stomach pain (red liquid)	None	Direct consumption	
Debark (Northern Ethiopia)	Fresh leaves	Treat fractures in livestock (horses, donkeys)	None	Topical application	
Kofele (Southeastern Ethiopia)	Roots	Relieve severe abdominal pain	None	Decoction	
Abaya District (Oromia)	Dried fruits	Treat roundworms (*Ascaris lumbricoides*)	Honey, local drink “Tella”	Decoction	Bekele and Reddy (2015)
Multiple regions in Ethiopia	Seeds	Expel tapeworms	Milk, honey, water	Decoction/oral liquid	Yirga et al. (2022)
Multiple regions in Ethiopia	Female flower powder	Prenatal care (reduce labor pain)	Barley porridge (Hordeum vulgare)	Oral paste	
Ambo (Western Ethiopia)	Leaves	Treat tapeworm infections	Water	Oral liquid	Tolossa (2021)

### 2.3 Future considerations

Women of the Amhara tribe are said to be unable to participate in the preparation of medicines during menstruation, as it is believed that this reduces the effectiveness of the medicines. In order to ensure the “purity” of the medicines, some groups restrict the preparation of medicines to unmarried women or menopausal persons. Although such taboos are not scientific, they objectively regulate traditional knowledge. However, with the development of modern medical technology, traditional medicines have been challenged by a new generation of synthetic drugs. Surveys show that only 12% of people under 30 years of age have mastered preparation techniques, and 89% of doctors over 60 years of age have a serious knowledge gap problem. To address this, we advocate for interdisciplinary collaborations with Ethiopian institutions to document practices digitally and integrate traditional medicine into biodiversity conservation efforts.

Information indicates that the natural forest of *H. abyssinica* in Ethiopia has declined by more than 60% during the period 2000–2020, the most important causes of which are excessive logging, agricultural expansion and climate change. Although the Forestry Law of 1994 clearly states that commercial logging is not allowed, poor enforcement has resulted in continued deforestation. Therefore, we propose establishing protected areas in Bale and Kofele regions and using ConPhyMP-compliant methods to standardize extract production, reducing pressure on wild populations.

## 3 Phytochemistry

**FIGURE 2:**
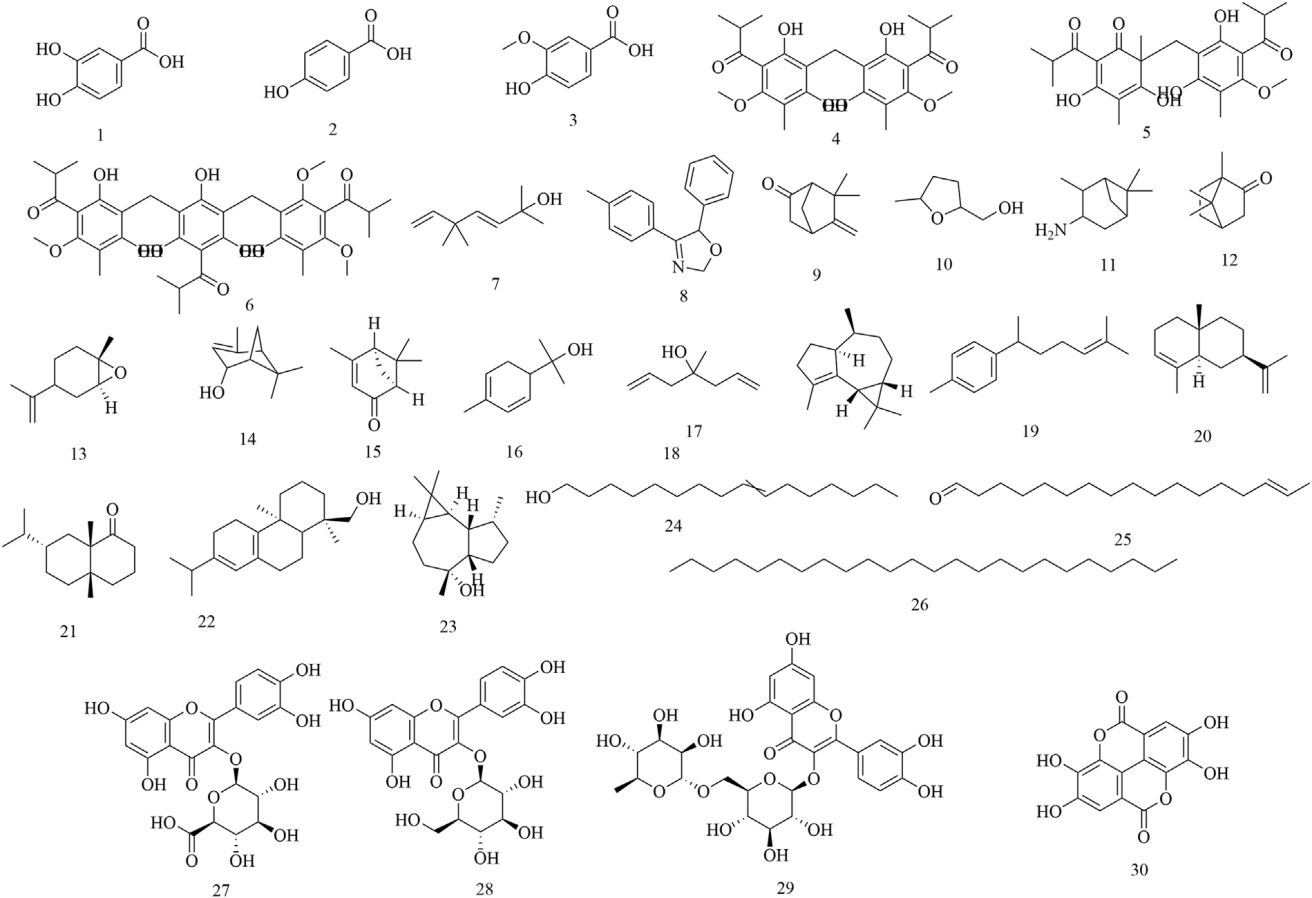
Chemical structures of secondary metabolites 1–30.

Up to now, a total of 95 secondary metabolites have been isolated and identified using NMR, MS and other chromatography techniques, including phenols, flavonoids, triterpenes and others. However, only 40% were characterized using advanced techniques (e.g., NMR/MS). Many studies relied on outdated methods (e.g., TLC), raising concerns about compound identification accuracy. The process of the discovery of these compounds is detailed as follows:

Initial phytochemical investigations concentrated on the flowers of *H. abyssinica*. In 1990, three phenolic compounds were characterized from *H. abyssinica* flowers using TLC, LC, UV, IR, MS and NMR spectroscopy, including protocatechuic acid (1), p-hydroxybenzoic acid (2) and vanillic acid (3) (Woldemariam et al., 1990). Subsequently, three phloroglucinol derivatives were systematically isolated from *H. abyssinica* flowers, which were identified as alpha-kosin (4), kosotoxin (5) and protokosin (6) (Woldemariam et al., 1992). In 2010, twenty compounds were characterized from the essential oils of *H. abyssinica* flowers using GLC/MS analysis, including yomogi alcohol (7), 2,5-dihydro-5-(4-methylphenyl)-4-phenyl-oxazole (8), 6-camphenone (9), tetrahydro-5-methyl-2-furanmethanol (10), 3-pinanylamine (11), L-camphor (12), trans-limonene oxide (13), verbenol (14), cis-Verbenone (15), α-phellandren-8-ol (16), diallyl methyl carbinol (17), gurjunene (18), curcumene (19), α-selinene (20), valeranone (21), palustrol (22), ledol (23), 9-hexadecen-1-ol (24), E-15-heptadecenal (25), and tetracosane (26). Of these, ledol (23) was the main chemical component, accounting for 58.57% (Nibret and Wink, 2010). In 2012, three flavonoids and one phenolic acid were systematically isolated from the flowers of *H. abyssinica* and they were identified as quercetin 3-O-β-glucuronide (27), quercetin 3-O-β-glucoside (28), rutin (29) and ellagic acid (30) (Thomsen, 2012) (Figure 2).

**FIGURE 3:**
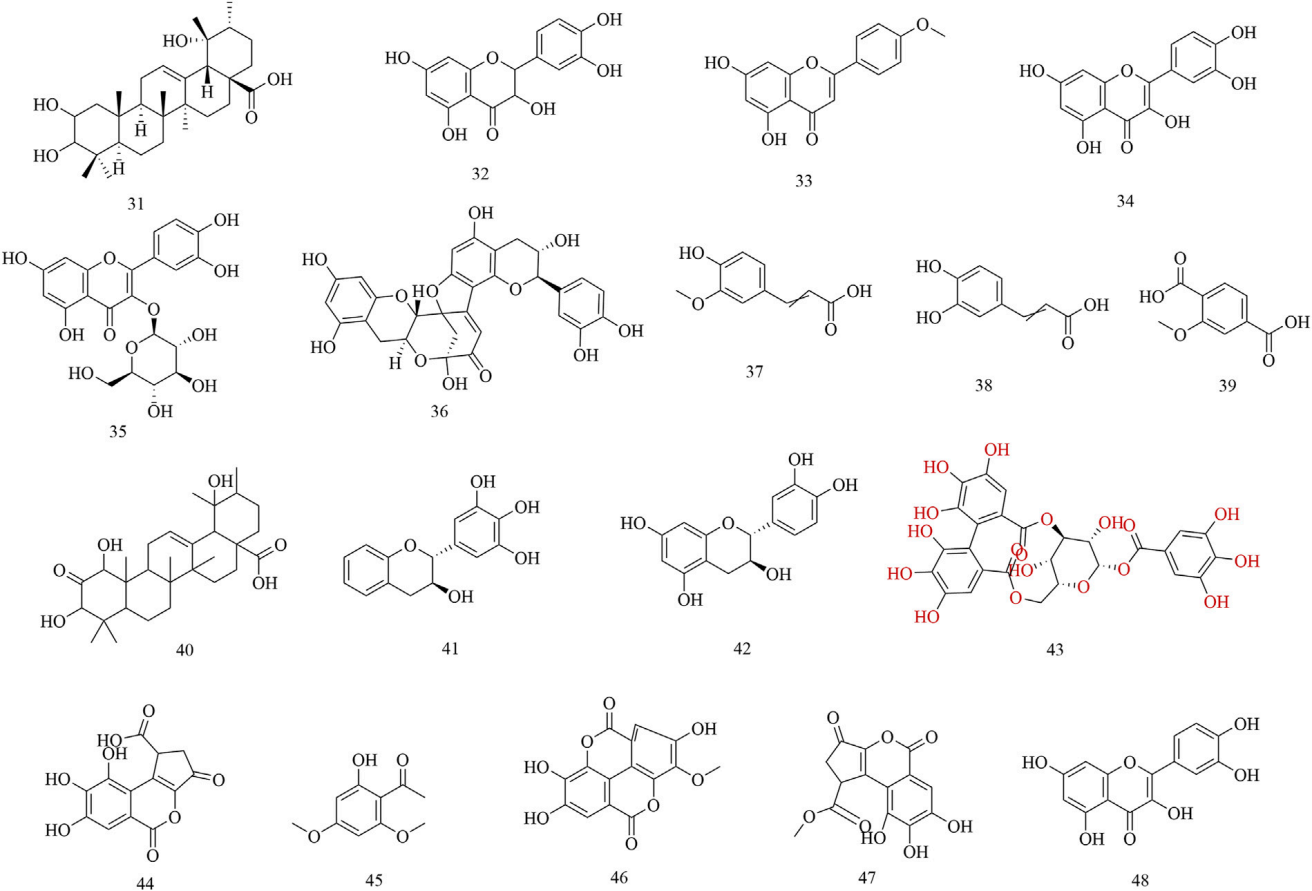
Chemical structures of secondary metabolites 31–48.

Subsequently, phytochemistry researchers isolated some secondary metabolites from the roots, leaves, and barks of *H. abyssinica*. For example, a pentacyclic triterpene, 2, 3, 19-trihydroxyurs-12-en-28-oic acid (31), was isolated from the leaves of *H. abyssinica* and its chemical structure was elucidated by one- and two-dimensional NMR. Five flavonoids, dihydroquercetin (32), acacetin (33), quercetin (34), isoquercitin (35), dehydrodicatechin A (36); and four phenolics, trans-ferulic acid (37), caffeic acid (38), protocatechuic acid (1), and 2-methoxyterephthalic acid (39); were systematically isolated from the roots of *H. abyssinica* (Fan et al., 2020). One triterpenoid, 1,3,19-trihydroxy-2-oxo-12-ursen-28-oic acid (40) and two flavans, 3,3′,4′,5′-tetrahydroxyflavan (41) and 3,3′,4′,5,7-pentahydroxyflavan (42) were separated from the barks of *H. abyssinica* and their chemical structures were elucidated by NMR and MS spectroscopy (Wagara et al., 2023). UPLC-MS coupled with molecular docking revealed compounds for the treatment of parasitic diseases, including corilagin (43), brevifolin carboxylic acid (44), brevifolin (45), methyl ellagic acid (46), methyl brevifolin carboxylate (47), and quercetin (48) (Fan et al., 2024) (Figure 3).

**FIGURE 4:**
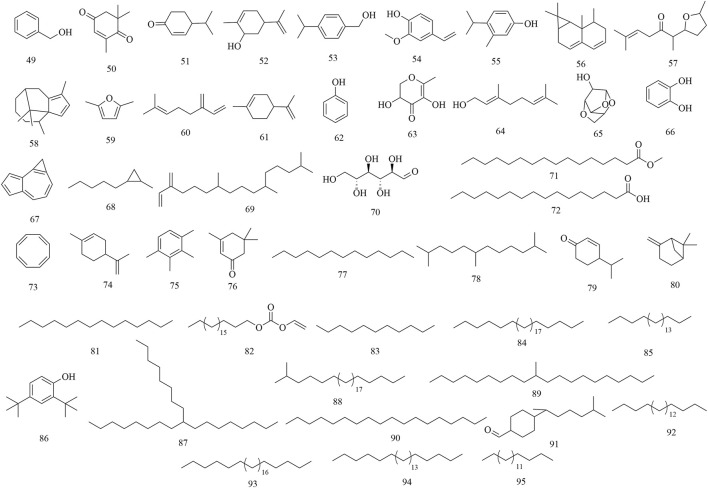
Chemical structures of secondary metabolites 49–95.

GC-MS analysis revealed the presence of major alkenones, alkanes, and phenols from the leaves of *H. abyssinica* (Kebie and Sisay, 2024). Among them, eleven compounds were characterized from its essential oils, which were presented as benzyl alcohol (49), L-camphor (12), 2,6,6-trimethyl-2-cyclohexene-1,4-dione (50), cryptone (51), cis-carveol (52), p-cymen-7-ol (53), 2-methoxy-4-vinylphenol (54), o-cymen-5-ol (55), aristolediene (56), cis-davanone (57), and cypera-2,4-diene (58). Of note, cis-davanone accounted for 14.73%, which was the primary presence. In addition, fifteen compounds were characterized from its methanol fraction, including 2,5-dimethylfuran (59), β-myrcene (60), limonene (61), phenol (62), L-camphor (12), 2,3-dihydro-3,5-dihydroxy-6-methyl-4H-pyran-4-one (63), geraniol (64), 1,4:3,6-dianhydro-alpha-d-glucopyranose (65), catechol (66), 1H-cycloprop[e]azulene (67), 1-methyl-2-pentylcyclopropane (68), neophytadiene (69), D-allose (70), methyl palmitate (71), and palmitic acid (72). Of these, β-myrcene (12.59%), neophytadiene (11.54%), and limonene (11.27%) were the major constituents. Furthermore, twenty-three compounds were characterized from its n-hexane fraction, including 1,3,5,7-cyclooctatetraene (73), limonene (74), prehnitene (75), isophorone (76), tridecane (77), 2,6,11-trimethyldodecane (78), 4-(1-methylethyl)-2-cyclohexen-1-one (79), β-pinene (80), tetradecane (81), eicosyl vinyl ester carbonic acid (82), dodecane (83), octacosane (84), eicosane (85), 2,4-di-tert-butylphenol (86), 9-octylheptadecane (87), 2-methyloctacosane (88), 10-methyleicosane (89), neophytadiene (69), octadecane (90), bisabolone (91), heneicosane (92), hexadecane (93), tetracosane (94), and heptadecane (95). Notably, 1,3,5,7-cyclooctatetraene was the main presence, accounting for 33.58% (Figure 4).

To evaluate the potential of these secondary metabolites as drug candidates, ADMET (Absorption, Distribution, Metabolism, Excretion, and Toxicity) analyses were performed using computational tools. Key findings are summarized in Table 2.

**TABLE 2:** ADMET analysis of key compounds from *H. abyssinica*.

Name	Absorption	Distribution	Metabolism	Excretion	Toxicity
Protocatechuic acid (1)	High (80%–90%)	Low	Glucuronidation	>70%	>5,000 mg/kg (rat)
α-Kosin (4)	Poor (<30%)	None	Hepatic phase I hydrolysis	85%	10 μM (MAC tumor cells)
Kosotoxin (5)	Poor (<25%)	None	CYP3A4 demethylation	80%	12 μM (MAC tumor cells)
Quercetin (34)	Moderate (60%–70%)	Low	CYP450-mediated oxidation	60%–75%	>2,000 mg/kg (mouse)
Caffeic acid (38)	High (85%–90%)	High (85%–90%)	Sulfation/Glucuronidation	>65%	>3,000 mg/kg (rat)

Note: ADMET predictions were performed using SwissADME (http://www.swissadme.ch) and ADMETlab 2.0 (https://admetmesh.scbdd.com/). Absorption (human oral bioavailability), Distribution (blood-brain barrier permeability), Metabolism (major pathways), Excretion (% renal), and Toxicity (LD_50_/IC_50_).

## 4 Pharmacology

### 4.1 Anthelmintic activity

**FIGURE 5:**
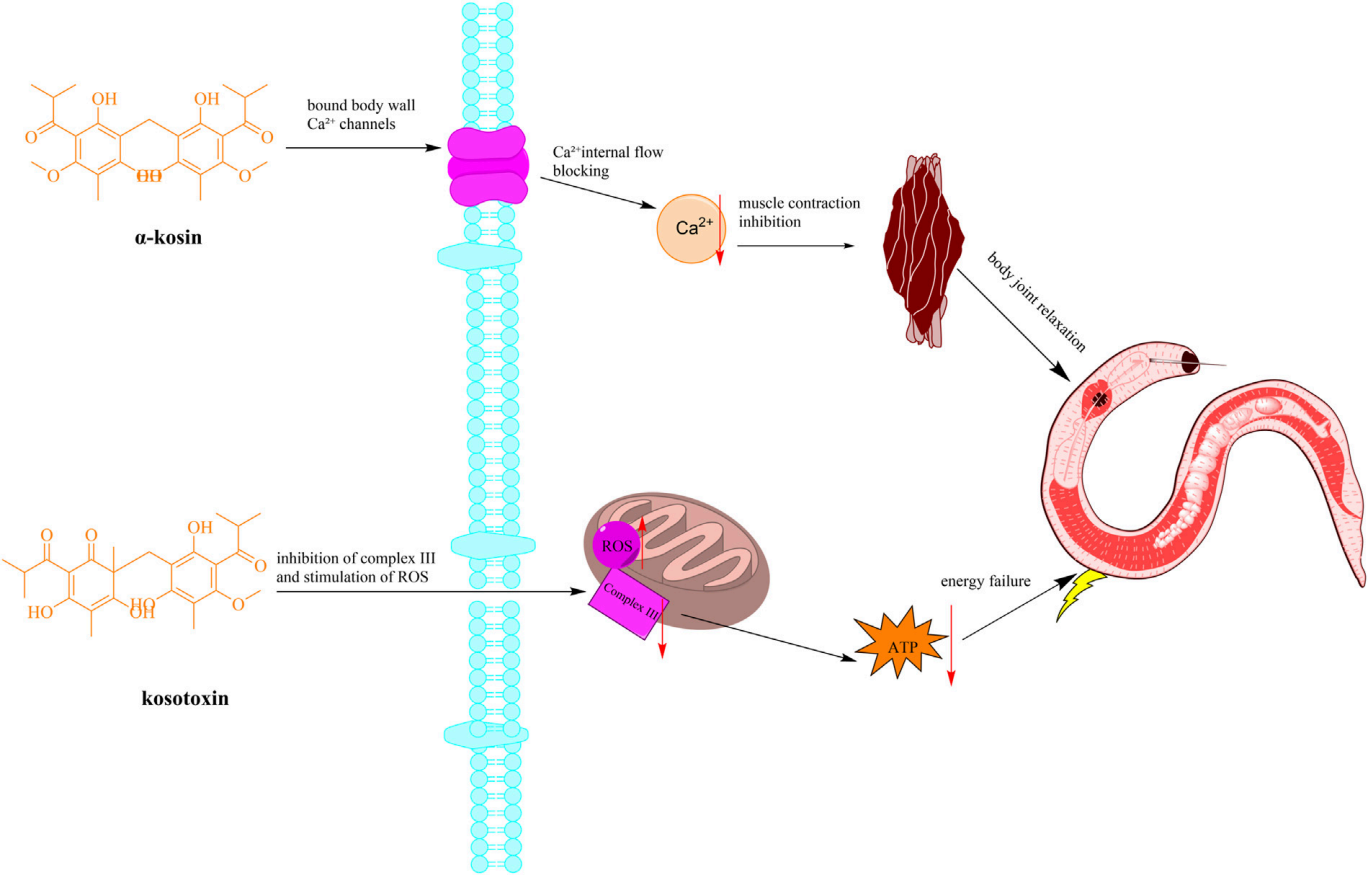
Schematic diagram of the mechanism of anthelmintic action of *H. abyssinica*. (Note: The active ingredient causes worm muscle paralysis (tapeworm segment contraction) and content leakage by interfering with parasite Ca^2+^ channels and inhibiting mitochondrial electron transport chain complex III).

The anthelmintic properties of *H. abyssinica* have been traditionally recognized by local communities. Recent scientific investigations have substantiated these claims through various experiments (Figure 5). However, most studies use crude extracts without specifying metabolite profiles, and mechanistic insights into parasite-target interactions are absent.

To start with, a study demonstrated that oral administration of *H. abyssinica* leaves (60 g/kg) to goats significantly reduced internal tapeworm egg counts (Abebe et al., 2000). However, this dosage regimen was empirically determined based on traditional practices, though further pharmacokinetic studies are needed to optimize therapeutic protocols. Since then, some studies have examined the methanol extracts from *H. abyssinica*. For instance, a research into the methanol extracts of *H. abyssinica* revealed that the methanolic extract of the stem bark (400 mg/kg) inhibited *Plasmodium berghei* by 83.33%, enhancing the mean survival time compared to a control group treated with distilled water (Belete and Orijino, 2019). The extract was prepared following standard protocols and compared to chloroquine (10 mg/kg) as positive control. Alemu et al. (2018) administered a methanolic flower extract (1,200 mg/kg, single dose) to Swiss albino mice infected with *Schistosoma mansoni*. The study reported an 84.57% reduction in fecal egg count compared to the negative control (distilled water), but lacked a positive control to benchmark efficacy. Additionally, the absence of a dose-response curve limits mechanistic interpretation (Alemu et al., 2018). Moreover, the methanolic extract of *H. abyssinica* stem barks (1 mg/mL) demonstrated a 67% mortality rate in the *Panagrellus redivivus* model, which was less effective compared to the 87% mortality observed in the levamisole group at 100 mM (Karumi et al., 2013). The model was selected based on its sensitivity to anthelmintics, though its clinical relevance for human helminths requires verification.

Additionally, the n-heptane, ethyl acetate, and methanol fractions of *H. abyssinica* flowers were evaluated for their efficacy against *Schistosoma mansoni*, *Clonorchis sinensis*, *Fasciola hepatica*, and *Echinostoma Caproni* using the microtitration plate method (Thomsen, 2012). The results indicated that the anthelmintic activity diminished with increasing polarity of the tested fractions.

### 4.2 Antimicrobial activity

*H. abyssinica* leaves were used as the main raw material. The Ag/ZnO/bentonite nanocomposite, synthesized from the aqueous extracts of *H. abyssinica* leaves, demonstrated a circle of inhibition of 17.3 ± 0.2 mm (10 mg.mL^-1^) against *E. coli*, with MIC of 156.25 μg.mL^-1^ and MBC of 312.5 μg.mL^-1^ (Zewudie et al., 2023). While Ag/ZnO nanoparticles showed antibacterial activity against *E. coli*, synthesis protocols lacked standardization, and cytotoxicity to mammalian cells was not assessed (Zewudie et al., 2023). Notably, the tested concentration exceeded typical antibiotic susceptibility ranges (1–2 mg/mL), potentially overestimating clinical relevance. The ZnO nanoparticle, biosynthesized from the aqueous extract of *H. abyssinica* leaves, exhibited a zone of inhibition of 21 ± 1.0 mm against *Staphylococcus epidermidis* (30 mg/mL) *via* the disc diffusion method (Zewde and Geremew, 2022). The silver nanoparticle, biosynthesized from the aqueous extract of *H. abyssinica* leaves, showed inhibition zone of 18.3 mm against *Salmonella typhimurium* (Melkamu and Bitew, 2021). The green-synthesized copper nanoparticles using the aqueous extract of *H. abyssinica* leaves was found to suppress the growth of *E. coli*, *Pseudomonas aeruginosa*, *Staphylococcus aureus*, and *Bacillus subtilis*, with zones of inhibition of 12.7, 12.7, 14.7, and 14.2 mm, respectively (Murthy et al., 2020). However, the absence of negative controls (e.g., unmodified nanoparticles) raises questions about specific phytochemical contributions. Most studies employed agar diffusion methods, which are inherently biased for nanomaterials due to variable diffusion kinetics; complementary microbroth dilution or time-kill assays are needed for robust activity quantification. A recent study synthesized magnesium oxide (MgO) nanoparticles using aqueous extracts from *H. abyssinica* flowers, demonstrating potent antibacterial activity against *E. coli* (15 ± 0 mm inhibition zone) and *S. aureus* (27 ± 0.28 mm). This highlights the plants potential in green nanotechnology for antimicrobial applications (Hirphaye et al., 2023).

The essential oil from *H. abyssinica* roots was found to show a higher mean of zone of inhibition (1.415 cm) than the leaves (1.14 cm) and the barks (0.908 cm) (Getachew and Getachew, 2019). The hexane, dichloromethane and methanol extracts of *H. abyssinica* leaves and barks were used to test their antimicrobial activity using the agar diffusion method, resulting in minimum inhibitory concentrations against *S. aureus*, MRSA and *P. aeruginosa* were ≤6.25 mg/mL (Ngeny et al., 2013). Polar solvent extracts showed reduced efficacy against gram-negative pathogens (*P. aeruginosa* MIC >6.25 mg/mL for methanol vs. 156.25 μg/mL for n-hexane fractions), suggesting lipophilic compounds may mediate membrane disruption (Ngeny et al., 2013). The dichloromethane and methanol extracts of stem bark of *H. abyssinica* showed zones of inhibition of 19 mm, 20 mm, and 18 mm against *S. aureus*, *E. coli* and *B. subtilis*, respectively (Karumi et al., 2013). In addition, the petroleum ether extract of the stem bark of *Hagenia abyssinica* against *S. aureus*, *E. coli*, and *B. subtilis*, with the zone of inhibition of 17 mm, 16 mm, and 16 mm, respectively (Karumi et al., 2013).

Currently, *H. abyssinica* nanomaterials have emerged as the key focus of research regarding antibacterial activity. While multiple studies report antibacterial activity of *H. abyssinica* nanomaterials, methodological limitations warrant cautious interpretation. In general, antimicrobial assays lack standardized controls, limiting translational relevance. For instance, agar diffusion protocols (6–8 mm disc vs. 10 mg/mL concentration variations) limits direct comparability between studies, underscoring the need for standardized antimicrobial assessment frameworks. In addition, non-polar fractions exhibited broader-spectrum activity (n-hexane MIC<156 μg/mL vs. methanol MIC>6.25 mg/mL). This result suggests lipophilic terpenoids mediate Gram-negative envelope disruption, but further compound-specific verification is required.

### 4.3 Antihyperglycemic and antidyslipidemic activities

The innovative application of *H. abyssinica* in the treatment of cardiovascular diseases bridges modern medicine with traditional practices. In the study of blood glucose, the aqueous fraction of *H. abyssinica* leaves (400 mg/kg) improved body weight in streptozotocin-induced diabetic mice, with a 44.10% decrease in blood glucose levels on day 14, compared to negative controls (Kifle et al., 2021d). Additionally, the methanolic extract of *H. abyssinica* leaves (400 mg/kg) reduced fasting blood glucose level by 43.53% in streptozotocin-induced diabetic mice (Kifle and Belayneh, 2020).

The methanolic extract of *H. abyssinica* leaves (500 μg/mL) demonstrated a 74.52% α-amylase inhibitory activity with an IC_50_ of 14.52 μg/mL; while the aqueous fraction of *H. abyssinica* leaves (500 μg/mL) showed a 62.54% α-amylase inhibition activity with an IC_50_ of 62.54% (Kifle et al., 2021b). While enzyme inhibition assays suggest mechanism of action, *in vivo* confirmation of target engagement is needed. Notably, the ethyl acetate fraction of *H. abyssinica* flowers (400 mg/kg) resulted in a 35.93% blood glucose level in streptozotocin-induced diabetic mice and a 54.23% α-amylase inhibitory activity at 800 μg/mL (Kifle et al., 2020). α-Amylase inhibition assays at supraphysiological concentrations (500 μg/mL) showed favorable IC_50_ values without enzyme kinetic analysis to determine inhibition type. These oversights limit translational potential; future studies should adhere to ARRIVE guidelines with proper controls and power calculations.

In the study of blood lipid, the aqueous fraction, ethyl acetate fraction and methanolic extract of *H. abyssinica* leaves (400 mg/kg) were found to significantly reduce serum total cholesterol, triglyceride, low-density lipoprotein cholesterol, cholesterol, and low-density lipoprotein cholesterol levels compared to negative controls (Kifle and Belayneh, 2020; Kifle et al., 2021d).

### 4.4 Antidiarrheal activity

*H. abyssinica* is frequently employed by local populations for the treatment of diarrhea, prompting further investigation by contemporary researchers. Studies have demonstrated that administering solvent fractions (aqueous, ethyl acetate, and chloroform) of *H. abyssinica* leaves (400 mg/kg) to mice with castor oil-induced diarrhea resulted in a prolonged stool frequency, a reduction in fecal weight, and a delayed onset of diarrhea when compared to negative controls (Kifle et al., 2021c). Gastrointestinal transit inhibition suggests antimotility mechanisms, though receptor binding assays are needed to confirm targets. In the same year, the methanolic extract of *H. abyssinica* leaves (400 mg/kg) reduced the volume and weight of intestinal contents in mice with castor oil-induced diarrhea compared to negative controls, exhibiting notable antiperistaltic effects (Kifle et al., 2021a).

### 4.5 Anti-oxidant activity

Oxidative free radical scavenging holds significant medical importance, offering protection to the cardiovascular and nervous systems, and aiding in immune regulation (Ionita, 2021; Jones, 2008). Presently, the DPPH method is predominantly utilized for *in vitro* research, with a notable gap in *in vivo* methodologies.

For example, the silver nanoparticle biosynthesized from the aqueous extract of *H. abyssinica* leaves (320 μg/mL) were found to possess 66% inhibition concentration via the DPPH method (Melkamu and Bitew, 2021). A DPPH assay revealed that the methanolic extract, water fraction, ethyl acetate fraction, and chloroform fraction of *H. abyssinica* leaves exhibited IC_50_ values of 10.25 μg/mL, 13.86 μg/mL, 16.34 μg/mL, and 18.83 μg/mL, respectively (Kifle et al., 2021b). The methanolic extract of *H. abyssinica* flowers (500 μg/mL) had 58.38% inhibition by DPPH method (Kifle et al., 2020). Caffeic acid was found to exhibit DPPH scavenging ability, with an IC_50_ of 7.858 μg/mL (Fan et al., 2020), highlighting the need to isolate individual antioxidants for mechanistic studies. While DPPH scavenging indicates free radical neutralization capacity, the clinical relevance requires validation in oxidative stress models like paraquat-induced hepatotoxicity.

### 4.6 Others

Pharmacological studies have demonstrated that *H. abyssinica* exhibits notable anti-tumor and anti-inflammatory properties. The MTT assay indicated that quercetin significantly inhibited cell growth in HT-29 and HepG2 cell lines. Additionally, n-hexane and ethyl acetate fractions derived from *H. abyssinica* roots (50 μg/mL) showed a higher inhibition rate against HepG2, SGC-7901, and HT-29 cell lines compared to other solvent fractions (Fan et al., 2020). In a carrageenan-induced mouse model of foot edema, administration of the methanol extract from *H. abyssinica* flowers (400 mg/kg) resulted in a 63.38% reduction in paw edema (Belachew et al., 2020). The above pharmacological effects of *H. abyssinica* were summarized in Table 3.

**TABLE 3:** The main pharmacological properties of *H. abyssinica*.

Pharmacological activities	Medicinal parts	Main findings	References
	Flowers	The anthelmintic activity of n-heptane and other fractions decreased with the increase in polarity	Thomsen (2012)
		The methanolic extract (1,200 mg/kg) significantly reduced the egg count and worm burden in mice and decreased the hepatic granuloma scores	Alemu et al. (2018)
	Leaves	The number of tapeworm eggs in goats was significantly reduced after oral administration	Abebe et al. (2000)
	Stem bark	The methanolic extract (1 mg/mL) had a 67% mortality rate in the model, which was lower than that of the comparison group	Karumi et al. (2013)
		The methanolic extract (1,200 mg/kg) significantly reduced the egg count and worm burden in mice and decreased the hepatic granuloma scores	Belete and Orijino (2019)
Antimicrobial activity	Flowers	The MgO nanoparticles synthesized had antibacterial activity against *Staphylococcus aureus* and *Escherichia coli*	Hirphaye et al. (2023)
	Leaves	The materials synthesized from the aqueous extract had antibacterial effect on *Escherichia coli*	Zewudie et al. (2023)
		The ZnO nanoparticles biosynthesized had antibacterial effect on *Staphylococcus epidermidis*	Zewde and Geremew (2022)
		The silver nanoparticles biosynthesized had antibacterial effect on *Salmonella typhimurium*	Melkamu and Bitew (2021)
		The copper nanoparticles synthesized inhibited the growth of several bacteria, with data of inhibition zones	Murthy et al. (2020)
	Leaves, bark	The various extracts had minimum inhibitory concentrations against *Staphylococcus aureus* and other bacteria less than or equal to 6.25 mg/mL	Ngeny et al. (2013)
	Roots	The mean of the inhibition zone of the essential oil was higher than that of leaves and barks	Getachew and Getachew (2019)
	Stem bark	The dichloromethane and other extracts had data of inhibition zones against *Staphylococcus aureus* and other bacteria	Karumi et al. (2013)
		The petroleum ether extract had data of inhibition zones against *Staphylococcus aureus* and other bacteria	Karumi et al. (2013)
Hypoglycemic and hypolipidemic activities	Flowers	The ethyl acetate extract reduced blood glucose and inhibited α-amylase in diabetic mice	Kifle et al. (2020)
	Leaves	The aqueous extract helped increase the body weight and reduce blood glucose in diabetic mice	Kifle et al. (2021d)
		The methanolic extract and others inhibited α-amylase	Kifle et al. (2021b)
		The methanolic extract reduced the fasting blood glucose in diabetic mice	Kifle and Belayneh (2020)
		Various extracts of leaves reduced the levels of multiple types of cholesterol in serum	Kifle and Belayneh (2020), Kifle et al. (2020)
Antidiarrheal activity	Flowers	The methanolic extract had a 58.38% inhibition rate by the DPPH method	Kifle et al. (2020)
	Leaves	Administration of solvent fractions changed the defecation frequency, fecal weight and the onset of diarrhea in mice with diarrhea	Kifle et al. (2021c)
		The methanolic extract reduced the volume and weight of intestinal contents in mice with diarrhea	Kifle et al. (2021a)
Antioxidant activity		The silver nanoparticles biosynthesized had a 66% inhibition concentration by the DPPH method	Melkamu and Bitew (2021)
		Various extracts of leaves had IC_50_ values by the DPPH method	Kifle et al. (2021a)
	Roots	Caffeic acid exhibited DPPH scavenging ability with an IC_50_ of 7.858 μg/mL	Fan et al. (2020)
Antitumor activity	Roots	The MTT assay showed that the extracts from roots inhibited the growth of multiple cell lines	Fan et al. (2020)
Anti-inflammatory activity	Flowers	The methanolic extract reduced paw edema by 63.38% in the mouse model of foot edema	Belachew et al. (2020)

## 5 Toxicity

The initial indications of *H. abyssinica* toxicity emerged in poultry farming. One study indicated that administering low daily doses of aqueous extract of *H. abyssinica* leaves (500 mg/kg) to chicks without significant adverse effects. On the contrary, higher doses (5,000 mg/kg) impaired their ability to detect moving objects in their peripheral vision and resulted in anatomical evidence of ganglion cell degeneration in the retina (Low, 1985). Notably, this toxicity was associated with oxidative stress markers and histological changes, though mechanistic details remain unclear. Therefore, further research is needed to validate these findings and explore dose-response relationships in different animal models.

**FIGURE 6:**
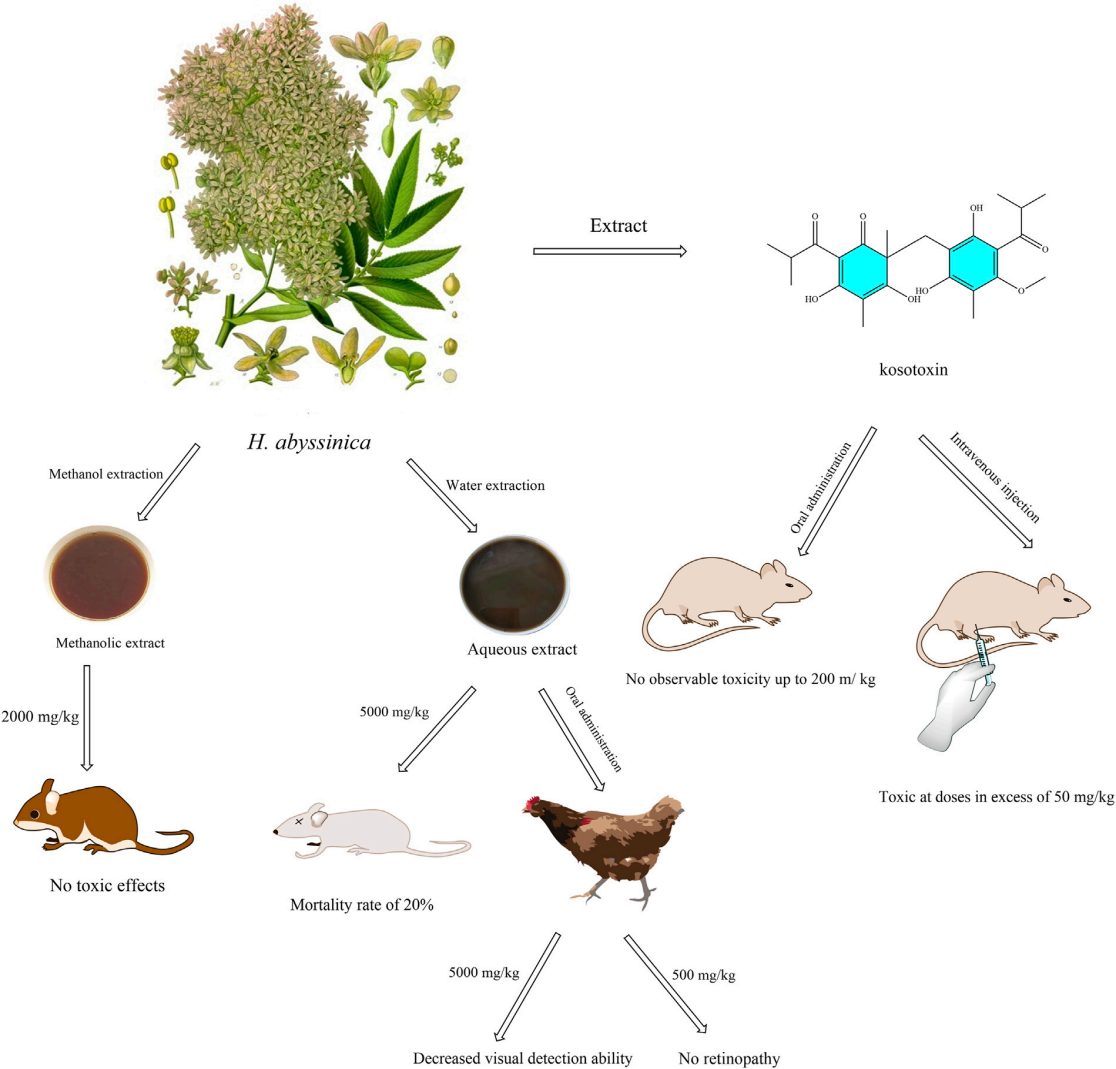
Illustration of the *in vivo* toxicity of extracts and compound of *H. abyssinica*. (This figure depicts the toxicity assessment of extracts (methanolic and aqueous extracts) and the compound kosotoxin of *H. abyssinica.* Mice were unaffected at a concentration of 2,000 mg/kg in the methanol extract, but mortality was 20% at 5,000 mg/kg in the aqueous extract. Chickens had reduced visual acuity at 5,000 mg/kg extract (no retinopathy at 500 mg/kg extract). Kosotoxin was not toxic to mice when administered orally at ≤200 mg/kg, but was toxic when administered intravenously at >50 mg/kg).

Currently, oral acute toxicity tests have been conducted on various extracts of *H. abyssinica*. For instance, the mice were given the methanolic extract of *H. abyssinica* leaves (2,000 mg/kg) for 14 days and the result revealed no observable signs of overt toxicity, as determined through physical and behavioral observations (Kifle et al., 2021c; Kifle et al., 2021d). Similarly, another acute toxicity investigation demonstrated that the methanolic extract of *H. abyssinica* flowers at the same dosage did not induce any toxic effects in mice (Kifle and Belayneh, 2020). These findings indicated the LD_50_ for *H. abyssinica* extracts exceeds 2,000 mg/kg. An *in vivo* acute toxicity study revealed that administering the aqueous extract of *H. abyssinica* leaves and stem barks to mice resulted in a 20% mortality rate at 5,000 mg/kg, indicating a safe dosage below this threshold (Ngeny et al., 2013). In a repeated administration study, oral administration of the aqueous extract of *H. abyssinica* flowers to rats showed no significant changes in body weight, biochemical parameters, or morphopathological conditions across all groups, suggesting a NOEL greater than 1,500 mg/kg. Furthermore, an *in vivo* study evaluating the active compound kosotoxin found no significant toxicity at oral doses of 200 mg/kg, whereas intravenous doses exceeding 50 mg/kg resulted in significant toxic reactions (Woldemariam et al., 1992) (Figure 6). However, most studies limited to 14-day observations, lacking teratogenicity assessments.

To summarize, acute oral toxicity tests show LD50 > 2,000 mg/kg for methanolic leaf and flower extracts in mice, with no adverse effects at 2,000 mg/kg over 14 days. Higher doses (5,000 mg/kg) caused retinal ganglion cell degeneration in chicks, highlighting optic nerve toxicity risks. However, chronic toxicity data are scarce, and few studies use omics-based approaches to identify toxic metabolites (e.g., kosotoxin). Standardized toxicology protocols following ConPhyMP guidelines are essential for safety assessment.

## 6 Conclusion

*H. abyssinica*, a traditional African medicine, is a highly exploitable primary healthcare medicine that can provide essential medical services to local populations. It is traditionally used to treat colds, stomach pains, and parasitic infections. At present, 95 compounds have been isolated and identified from *H. abyssinica*, including terpenoids, flavonoids, and phenols. These secondary metabolites might be responsible for its pharmacological properties such as anthelmintic, antibacterial, lipid-lowering and antioxidant. In addition, oral acute toxicity tests revealed the safe dose of 2,000 mg/kg *in vivo* without obvious adverse effects. *H. abyssinica* possesses significant medical relevance, which highlight its potential as a source of therapeutic agents warranting further scientific investigation.

## 7 Further perspectives

### 7.1 Phytochemical insight

Discovering bioactive compounds on the basis of the development of modern science and technology is the key to the research and development of new medicines (Yuan et al., 2016; Zhang et al., 2023). On the one hand, the identified active constituents can be used as potential lead or target compounds for the synthesis of medicines. On the other hand, these chemical compounds form the foundation for pharmacological studies on action mechanisms.

However, the metabolites contained in *H. abyssinica* are complex, and the present discoveries are still insufficient, necessitating the use of advanced separation techniques for further identification. The use of supercritical fluid, microwave, ultrasound and other modern extraction techniques instead of the traditional hydroalcoholic extraction method can further improve the extraction efficiency of the active components of traditional medicine (Krakowska-Sieprawska et al., 2022; Raynie, 2004). The LC-MS-NMR coupling technology provides a powerful means for the comprehensive and precise identification of pharmacodynamic compounds (Gathungu et al., 2020). The implementation of this technology will provide theoretical basis for the synthesis, modification and conformational relationship of natural compounds, and promote the research and development of new medicines.

Current phytochemical analyses rely on traditional HPLC-UV and GC-MS, but multi-omics approaches (e.g., metabolomics) and network pharmacology could uncover synergistic metabolite interactions. Based on the “multi-component-multi-target” pharmacodynamic characteristics of botanical drug, it is of great significance to establish the network model of “component-target-disease” for the prediction of pharmacodynamic substances and targets (Li et al., 2023; Li et al., 2022). The establishment of a complete database of chemical constituents of *H. abyssinica* will be a valuable reference for exploring pharmacodynamic component groups.

### 7.2 Pharmacological insight

The nanomaterials synthesized from *H. abyssinica*, which can directly interact with cells and help to improve the bioavailability and enhance the therapeutic efficacy, is an interesting research direction. Particular attention needs to be paid to the biodistribution of the nano-agents, as none of the current synthetic materials are radiolabeled for tracking across the blood-brain/placental barrier. PET-CT combined with 68Ga isotope labeling is recommended for *in vivo* distribution studies.

Current research on *H. abyssinica* nanomaterials is still focused on bacterial inhibition. Future studies should optimize NP surface functionalization for target specificity (e.g., tumor-homing ligands) and co-delivery of bioactive compounds (e.g., quercetin and kosotoxin) to synergize anti-infective and anticancer effects (Fan et al., 2024). Previous pharmacological activities are reported very naively, while the molecular level has not been studied deeply enough. Link phytochemicals to molecular targets via relevant knockout models and *in silico* docking studies with experimentally validated protein structures. Therefore, research hotspots of the interaction between pharmaceutical molecules and target proteins, the effect on intracellular signaling pathways, and the regulation of gene expression, need to be carried out in the future (Harrison et al., 2022; Huang et al., 2025; Tansaz and Tajadini, 2016; Wang et al., 2018).

The introduction of pharmacokinetic studies in traditional medicines can better evaluate the digestion and absorption process of active molecules in the body, which is the key to influence the efficacy and toxicity. It is important to replace arbitrary extract concentrations with pharmacokinetic-guided dosing and adopt organ-on-chip models to mimic human absorption. In addition, traditional pharmacology needs to be integrated with clinical practice to verify the effectiveness and safety of medicines. For instance, for prioritized indications such as tapeworm eradication, need to be validated via randomized controlled trials (RCTs), accompanied by strict phytochemical standardization where marker compounds are quantified by HPLC.

### 7.3 Toxicological insight

The commonly used methods of acute toxicity studies are limited to short-term effects and lack studies on distant and long-term toxicity. On this basis, the basic principles and methods of toxicology are comprehensively applied to evaluate the toxicity of traditional medicines, with a view to constructing a set of pharmacodynamic substance basis and safety evaluation model that meets the clinical reality. For instance, omics technologies (proteomics, metabolomics) can be used to identify early toxicity biomarkers (e.g., liver/kidney injury markers) to establish safe therapeutic windows, as demonstrated in subchronic hepatic toxicity studies (Kifle et al., 2021a). Perhaps in the future, genomics, proteomics, metabolomics and other methods should be utilized to explore the mechanism of toxicity at multiple levels, such as molecular, cellular, tissue and organ, and to clarify the basis of the toxic substances, the target of action and the signaling pathway (Lu et al., 2022; Pang et al., 2020).

## Collaboration statement

While this review synthesizes data from Ethiopian and international studies, the authors acknowledge the need for stronger collaboration with researchers from Ethiopia and neighboring countries to ensure contextual accuracy and cultural relevance. Future work will prioritize such partnerships.

## Abbreviations

ADMET, Absorption, Distribution, Metabolism, Excretion, and Toxicity; CC_50_, Cubic Centimeter; DPPH, 1,1-Diphenyl-2-picrylhydrazyl; IC_50_, Half maximal inhibitory concentration; IR, Infrared spectrum; LC, Liquid Chromatography; LD_50_, Median lethal dose; MIC, Minimum Inhibitory Concentration; MS, Mass Spectrometry; NMR, Nuclear Magnetic Resonance; UPLC, Ultra Performance Liquid Chromatography; UV, Ultraviolet spectrum.
